# On combinational contractions with applications

**DOI:** 10.1016/j.heliyon.2025.e41905

**Published:** 2025-01-14

**Authors:** Mohammed Shehu Shagari, Manzuma Mustapha, Hala H. Taha, Sarah Aljohani, Nabil Mlaiki

**Affiliations:** a Department of Mathematics, Faculty of Physical Sciences, Ahmadu Bello University, Zaria, Nigeria; b Department of Mathematical Sciences, College of Science, Prince Nourah bint Abdulrahman University, P. O. Box 84428, Riyadh, Saudi Arabia; c Department of Mathematics and Sciences, Prince Sultan University, Riyadh 11586, Saudi Arabia

**Keywords:** Fixed point, Jaggi contraction, Meir-Keeler contraction, Zamfirescu contraction, Fredholm nonlinear

## Abstract

The aim of this paper is to provide a new hybrid contraction that is a combination of Jaggi, Meir-Keeler and Zamfirescu-type contractions in the framework of a complete metric space. The existence and uniqueness conditions of a fixed point of such contractions are investigated. An example is constructed to demonstrate the validity of the obtained results. Furthermore, the presented result is utilized to formulate new criteria for solving a class of nonlinear Fredholm integral equation.

## 1 Introduction

Over hundred years ago, Banach [2] published what is known as the first metric fixed point theorem. Before Banach, notable mathematicians such as Picard and Liouville had employed the fixed point method to tackle specific differential equations, particularly initial value problems. Drawing inspiration from their work, Banach viewed it as a distinct and independent result situated within the contexts of nonlinear functional analysis and point-set topology. The metric fixed point theorem has since attracted the attention of various researchers. The key reason for their interest in investigating metric fixed point theory lies in its natural and significant connection between theoretical findings in nonlinear functional analysis and their applications in the sciences. One of these extensions was presented by Meir-Keeler [10], in what is currently called the Meir-Keeler contraction or the uniform contraction. Later after, more than a handful of variants of uniform contractions have been presented. Not long ago, Kumar et al. [11] refined the idea in [10] by introducing the concept of contravariant (α−ψ)-Meir-Keeler contractive mappings in bipolar metric spaces. In a related result, Singh et al. [18] studied a hybrid concept involving the Meir-Keeler and Khan contractions and discussed some existence criteria of fixed points for such mappings. Jiddah et al. [8] came up with a new technique to define a family of contractions of Jaggi-type in *G*-metric space and proved some fixed point theorems that cannot be followed from the existing ones. The earliest version of the contraction mapping principle involving rational equality was due to Jaggi [7]. And, one of the well known results along this direction is the Zamfirescu fixed point theorem (see [15]).

Following the above development, we noticed that a hybrid concept underlying the main ideas of Banach [2], Jaggi [7], Meir-Keeler [10] and Zamfirescu [15] has not been adequately examined. Hence, this paper introduces a novel contraction, under the name combinational contraction. Conditions for the existence of fixed points are formulated and discussed. A comparative example is constructed to support the assumptions forming our main idea. From application perspective, one of our obtained results is employed to establish new criteria for solving nonlinear Fredholm integral equation.

This paper commences with an introductory overview and literature review in Section 1. Section 2 outlines the fundamental results and mathematical notations used in this work. Section 3 discusses the key findings and some special cases of the obtained fixed point results. Using one of the acquired consequences herein, the existence and distinctiveness of solutions of certain nonlinear integral equations is investigated in Section 4. While in Section 5, conclusions are given.

## 2 Preliminary

In this section, basic definitions and results that are relevant to the presentation of our ideas are collected. We say that ψ:[0,∞)⟶[0,∞) is a (c)-comparison function (see [3], [14]) if it is nondecreasing and fulfills: there are $l_{\circ }\in N$ and k∈(0,1) and a convergent series $\sum_{l=1}^{\infty }z_{l}$ such that $z_{l}\geq 0$ and

$$\psi^{l+1}(t)\leq k\psi^{l}(t)+z_{l},$$

for $l\geq l_{\circ }$ and t≥0.

The set of all (c)- comparison functions will be denoted by Ψ. Every ψ∈Ψ verifies the following [14]:

(a) ψ(z)<z, for any $z\in R^{+}$;
(b) *ψ* is continuous at 0;
(c) the series $\sum_{l=1}^{\infty }\psi^{\prime }(z)$ is convergent for z≥0.

Definition 2.1 [10] A mapping Ϝ:(S,λ)⟶(S,λ) is termed a Meir-Keeler contraction on *S*, if for every ε>0, we can find δ>0 such that

(2.1) ε≤λ(Y,Z)<ε+δimpliesλ(ϜY,ϜZ)<ε,

for every Y,Z∈S.

Theorem 2.2 *[10]**Any Meir-Keeler contraction*Ϝ:(S,λ)⟶(S,λ)*admits a unique fixed point.*

In a recent study, Bisht and Rackocevic [4] extended the uniform contraction notion in the following manner.

Theorem 2.3 *[4]**Suppose a mapping*Ϝ:(S,λ)⟶(S,λ)*meets the following requirements:*

*(1)* *for a given*ε>0*, we can find a*δ(ε)>0*such that*
  ε<M(Y,Z)<ε+δ(ε)impliesλ(ϜY,ϜZ)≤ε;
*(2)* λ(ϜY,ϜZ)<M(Y,Z)*whenever*M(Y,Z)<0*; for any*Y,Z∈S*, where*

$$M(Y,Z)=\max\{\lambda (Y,Z),\sigma \lambda (Y,ϜY)+(1-\sigma )\lambda (Z,ϜZ),(1-\sigma )\lambda (Y,ϜY)+\sigma \lambda (Z,ϜZ),\frac{\beta [\lambda (Y,ϜZ)+\lambda (Z,ϜY)]}{2}\}.$$

Definition 2.4 [15] Let (S,λ) be a metric space. A mapping Ϝ:S⟶S is referred to as a Zamfirescu contraction if for all Y,Z∈S,

$$\lambda (ϜY,ϜZ)<\max\{\lambda (Y,Z),\frac{1}{2}[\lambda (Y,ϜY)+\lambda (Z,ϜZ)],\frac{1}{2}[\lambda (Y,ϜZ)+\lambda (Z,ϜY)\}.$$

Definition 2.5 [13] Let ρ:S×S⟶[0,+∞) be a mapping, where S≠0. A self mapping Ϝ:(S,λ)⟶(S,λ) is called triangular *ρ*-orbital admissible and denoted as $Ϝ\in T_{S}^{\rho }$ if

$$\rho (Y,ϜY)\geq 1\;\mathrm{implies}\;\rho (ϜY,{Ϝ}^{2}Y)\geq 1,$$

and

ρ(Y,Z)≥1,andρ(Z,ϜZ)≥1,implyρ(Y,ϜZ)≥1,

for all Y,Z∈S.

This concept was used by many researchers, in order to prove variant fixed point results (e.g., see [1], [6], [9]).

## 3 Main results

We begin this section by defining a new contraction, termed as a combinational contraction. Let (S,λ) be a metric space. Take into account the mapping Ϝ:S⟶S and the set of fixed points, Fix(S)={Y∈S:ϜY=Y}. We define the expression $M_{Ϝ}^{\varsigma }(Y,Z)$ as follows:

$$M_{Ϝ}^{\varsigma }(Y,Z)=\left\{ \begin{array}{c} {\left[\beta_{1}{\left(\frac{\lambda (Y,ϜY)\lambda (Z,ϜZ)}{\lambda (Y,Z)}\right)}^{\varsigma }+\beta_{2}{\left(\lambda (Y,Z)\right)}^{\varsigma }\right]}^{\frac{1}{\varsigma }},\\ \;\;\;\;\;if\;\varsigma >0,\;Y,Z\in S,Y\neq Z;\\ {\left(\lambda (Y,ϜY)\right)}^{\beta_{1}}{\left(\lambda (Z,ϜZ)\right)}^{\beta_{2}},\\ \;\;\;\;\;if\;\varsigma =0,\;Y,Z\in S, \end{array}\right.$$

where $\beta_{i}\geq 0,i=1,2,3$ are such that $\beta_{1}+\beta_{2}+\beta_{3}=1$.

Definition 3.1 Let (S,λ) be a metric space. A mapping Ϝ:S⟶S is referred to as a combinational contraction if for every distinct Y,Z∈S and for all ε>0, we can find a δ>0 such that

(3.1) $$\varepsilon <\max\{\lambda (Y,Z),\frac{1}{2}[\lambda (Y,ϜY)+\lambda (Z,ϜZ)],\;\frac{1}{2}[\lambda (Y,ϜZ)+\lambda (Z,ϜY)],M_{Ϝ}^{S}(Y,Z)\}<\varepsilon +\delta$$

(3.2) ⇒ρ(Y,Z)λ(ϜY,ϜZ)≤ε.

The next Lemma is easily obtained from (3.1).

Lemma 3.2 *Let*(S,λ)*be a metric space. If*Ϝ:S⟶S*is a combinational contraction on S, then*

(3.3) $$\rho (Y,Z)\lambda (ϜY,ϜZ)<\max\{\lambda (Y,Z),\frac{1}{2}[\lambda (Y,ϜY)+\lambda (Z,ϜZ)],\;\frac{1}{2}[\lambda (Y,ϜZ)+\lambda (Z,ϜY)],M_{Ϝ}^{\varsigma }(Y,Z)\}.$$

Theorem 3.3 *Let*(S,λ)*be a complete metric space and*Ϝ:S⟶S*be any continuous combinational contraction on S, then Ϝ has a fixed point in S if we can find*$Y_{\circ }\in S$*such that*$\rho (Y_{\circ },ϜY_{\circ })\geq 1$*and*$\rho (Y_{\circ },{Ϝ}^{2}Y_{\circ })\geq 1$*.*

Proof Let $Y_{\circ }\in S$ represent any chosen fixed element. We establish the sequence $\left\{Y_{m}\right\}$ in the following way:

$$Y_{m}=ϜY_{m-1}={Ϝ}^{m}Y_{\circ },$$

for all m∈N and assume that $\lambda (Y_{m},Y_{m+1})>0$, for all n∈N⋃{0}. Indeed, if for some $i_{\circ }\in N⋃\{0\}$, we have $\lambda (Y_{i_{\circ }},Y_{i_{\circ }+1})=0$, it follows that $Y_{i_{\circ }}=Y_{i_{\circ }+1}=ϜY_{i_{\circ }}$. Thus, $Y_{i\circ }$ is a fixed point of the mapping *Ϝ* and the proof is complete. Since the mapping *Ϝ* is triangular *ρ*-admissible by assumption, it follows that

$$\rho (Y_{\circ },ϜY_{\circ })\geq 1\Rightarrow \rho (Y_{1},Y_{2})=\rho (ϜY_{\circ },{Ϝ}^{2}S_{\circ })\geq 1\Rightarrow ...$$

(3.4) $$\Rightarrow \rho (Y_{n},Y_{n+1})\geq 1,$$

for every n∈N. We shall study two cases; these are ς>0 and ς=0.

**Case (A):** For the case ς>0, letting $Y=Y_{n-1}$ and $Z=Y_{n}=ϜY_{n-1}$ in (3.3), we obtain

(3.5) $$\lambda (Y_{n},Y_{n+1})\leq \rho (Y_{n-1},Y_{n})\lambda (ϜY_{n-1},ϜY_{n})<\max\{\lambda (Y_{n-1},Y_{n}),\frac{1}{2}[\lambda (Y_{n-1},ϜY_{n-1})+\lambda (Y_{n},ϜY_{n})],\frac{1}{2}[\lambda (Y_{n-1},ϜY_{n})+\lambda (Y_{n},ϜY_{n-1})],M_{Ϝ}^{\varsigma }(Y_{n-1},Y_{n})\}<\max\{\lambda (Y_{n-1},Y_{n}),\frac{1}{2}[\lambda (Y_{n-1},Y_{n})+\lambda (Y_{n},Y_{n+1})],\;\;\;\frac{1}{2}[\lambda (Y_{n-1},Y_{n+1})+\lambda (Y_{n},Y_{n})],M_{Ϝ}^{\varsigma }(Y_{n-1},Y_{n})\}<\max\{\lambda (Y_{n-1},Y_{n}),\frac{1}{2}[\lambda (Y_{n-1},Y_{n})+\lambda (Y_{n},Y_{n+1})],\;\;\;\frac{1}{2}[\lambda (Y_{n-1},Y_{n+1})],M_{Ϝ}^{\varsigma }(Y_{n-1},Y_{n})\},$$

where

$$M_{Ϝ}^{\varsigma }(Y_{n-1},Y_{n})={\left[\beta_{1}{\left(\frac{\lambda (Y_{n-1},ϜY_{n-1}),\lambda (Y_{n},ϜY_{n})}{\lambda (Y_{n-1},Y_{n})}\right)}^{\varsigma }+\beta_{2}{\left(\lambda (Y_{n-1},Y_{n})\right)}^{\varsigma }\right]}^{\frac{1}{\varsigma }}={\left[\beta_{1}{\left(\frac{\lambda (Y_{n-1},Y_{n}),\lambda (Y_{n},Y_{n+1})}{\lambda (Y_{n-1},Y_{n})}\right)}^{\varsigma }+\beta_{2}{\left(\lambda (Y_{n-1},Y_{n})\right)}^{\varsigma }\right]}^{\frac{1}{\varsigma }}\leq {\left[\beta_{1}{\left(\lambda (Y_{n},Y_{n+1})\right)}^{\varsigma }+\beta_{2}{\left(\lambda (Y_{n-1},Y_{n})\right)}^{\varsigma }\right]}^{\frac{1}{\varsigma }}.$$

If we can find $n_{\circ }\in N$ such that $\lambda (Y_{n_{\circ }},Y_{n_{\circ }+1})\geq \lambda (Y_{n_{\circ }-1},Y_{n})$, then

$$M_{Ϝ}^{\varsigma }(Y_{n_{\circ }-1},Y_{n_{\circ }})\leq {\left[\beta_{1}{\left(\lambda (Y_{n_{\circ }},Y_{n_{\circ }+1})\right)}^{\varsigma }+\beta_{2}{\left(\lambda (Y_{n_{\circ }},Y_{n_{+}1})\right)}^{\varsigma }\right]}^{\frac{1}{\varsigma }}={\left[\beta_{1}+\beta_{2}\right]}^{\frac{1}{\varsigma }}\lambda (Y_{n_{\circ }},Y_{n+1})=\lambda (Y_{n_{\circ }},Y_{n_{\circ }+1}).$$

Then, $\max\{\lambda (Y_{n_{\circ }},Y_{n_{\circ }+1}),\lambda (Y_{n_{\circ }},Y_{n_{\circ }+1}),\frac{1}{2}[\lambda (Y_{n_{\circ }-1},Y_{n_{\circ }+1})],M_{Ϝ}^{\varsigma }(Y_{n_{\circ }-1},Y_{n_{\circ }})\}=\lambda (Y_{n_{\circ }},Y_{n_{\circ }+1})$. Using (3.4) and (3.5), we get

$$\lambda (Y_{n_{\circ }},Y_{n_{\circ }+1})\leq \rho (Y_{n_{\circ }-1},Y_{n_{\circ }})\lambda (ϜY_{n_{\circ }-1},ϜY_{\circ })<\max\{\lambda (Y_{n_{\circ }},Y_{n_{\circ }+1}),\lambda (Y_{n_{\circ }},Y_{n_{\circ }+1}),\frac{1}{2}[\lambda (Y_{n_{\circ }-1},Y_{n_{\circ }+1})],\;\;\;\;\;\;M_{Ϝ}^{\varsigma }(Y_{n_{\circ }-1},Y_{n_{\circ }})\}\leq \lambda (Y_{n_{\circ }},Y_{n_{\circ }+1}),$$

which is a contradiction. Therefore, $\lambda (Y_{n},Y_{n+1})<\lambda (Y_{n-1},Y_{n})$ for all n∈N and (3.5) becomes

$$\lambda (Y_{n},Y_{n+1})<\lambda (Y_{n-1},Y_{n}),$$

for all n∈N. As a result, we can find l≥0 such that $\lim_{n\to +\infty }\lambda (Y_{n-1},Y_{n})=l$. If l>0, then,

$$\lambda (Y_{m},Y_{m+1})\geq l>0,$$

for any m∈N. On the one hand, since Lemma 3.2 holds for every given ε>0, in particular, we can choose ε=l and let δ>0 be such that Lemma 3.2 is fulfilled. However, since also $\lim_{n\to +\infty }\max\{\lambda (Y_{n-1},Y_{n}),\frac{1}{2}[\lambda (Y_{n-1},Y_{n+1})+\lambda (Y_{n},Y_{n+1})],\frac{1}{2}[\lambda (Y_{n-1},Y_{n+1})],M_{Ϝ}^{\varsigma }(Y_{n-1},Y_{n})\}=l$, we can find $m_{\circ }\in N$ such that

$$l<\max\{\lambda (Y_{m_{\circ }-1},Y_{m_{\circ }}),\frac{1}{2}[\lambda (Y_{m_{\circ }-1},Y_{m_{\circ }+1})+\lambda (Y_{m_{\circ }},Y_{m_{\circ }+1})],\frac{1}{2}[\lambda (Y_{m_{\circ }-1},Y_{m_{\circ }+1})],M_{Ϝ}^{\varsigma }(Y_{m_{\circ }-1},Y_{m_{\circ }})\}<l+\delta .$$

Thus, by (3.2) together with (3.4), we obtain

$$\lambda (Y_{m_{\circ }},Y_{m_{\circ }+1})\leq \rho (Y_{m_{\circ }},Y_{m_{\circ }+1})\lambda (ϜY_{m_{-}1},ϜY_{m_{\circ }})<l,$$

which is a contradiction. Therefore,

(3.6) $$\lim_{n\to +\infty }\lambda (Y_{n},Y_{n+1})=l=0.$$

We now assert that the sequence $\left\{Y_{n}\right\}$ is Cauchy. Assuming ε>0, we may determine $\delta^{\prime }=\min\{\delta (\varepsilon ),\varepsilon ,1\}$. Therefore, it follows from (3.6) that we can find $j_{\circ }\in N$ such that

(3.7) $$\lambda (Y_{n},Y_{n+1})<\frac{\delta^{\prime }}{2},$$

for all $n\geq j_{\circ }$. We now examine the set

(3.8) $$A=\{Y_{k}|k\geq j_{\circ },\lambda (Y_{k},Y_{j_{\circ }})<\varepsilon +\frac{\delta^{\prime }}{2}\}.$$

Suppose that ϜZ∈A whenever $Z=Y_{k}\in A$. Indeed, in the case of $k=j_{\circ }$, we have $ϜY_{k}=ϜY_{j_{\circ }}=Y_{j_{\circ }+1}$ and taking (3.7) into consideration, leads to

(3.9) $$\lambda (Y_{j_{\circ }},Y_{j_{\circ }+1})<\frac{\delta^{\prime }}{2}<\varepsilon +\frac{\delta^{\prime }}{2}.$$

As a result, presume that $k>j_{\circ }$ and identify two scenarios, specifically:

**Case (1):** Assume that

(3.10) $$\varepsilon <\lambda (Y_{k},Y_{j_{\circ }})<\varepsilon +\frac{\delta^{\prime }}{2}.$$

Then,

$$M_{Ϝ}^{\varsigma }(Y_{k},Y_{j_{\circ }})={\left[\beta_{1}{\left(\frac{\lambda (Y_{k},ϜY_{k}),\lambda (Y_{j_{\circ }},ϜY_{j_{\circ }})}{\lambda (Y_{k},ϜY_{j_{\circ }})}\right)}^{\varsigma }+\beta_{2}{\left(\lambda (Y_{k},Y_{j_{\circ }})\right)}^{\varsigma }\right]}^{\frac{1}{\varsigma }}={\left[\beta_{1}{\left(\frac{\lambda (Y_{k},Y_{k+1}),\lambda (Y_{j_{\circ }},Y_{j_{\circ }+1})}{\lambda (Y_{k},Y_{j_{\circ }+1})}\right)}^{\varsigma }+\beta_{2}{\left(\lambda (Y_{k},Y_{j_{\circ }})\right)}^{\varsigma }\right]}^{\frac{1}{\varsigma }}<{\left[\beta_{1}{\left(\lambda (Y_{k},Y_{k+1}\right)}^{\varsigma }+\beta_{2}{\left(\lambda (Y_{k},Y_{j_{\circ }})\right)}^{\varsigma }\right]}^{\frac{1}{\varsigma }}<{\left[\beta_{1}{\left(\frac{\delta^{\prime }}{2}\right)}^{\varsigma }+\beta_{2}{\left(\varepsilon +\frac{\delta^{\prime }}{2}\right)}^{\varsigma }\right]}^{\frac{1}{\varsigma }}\leq {\left(\beta_{1}+\beta_{2}\right)}^{\frac{1}{\varsigma }}(\frac{\delta^{\prime }}{2}+\varepsilon +\frac{\delta^{\prime }}{2})\leq {\left(\beta_{1}+\beta_{2}\right)}^{\frac{1}{\varsigma }}(\varepsilon +\delta^{\prime })\leq \varepsilon +\delta^{\prime }.$$

In this instance,

$$\varepsilon <\lambda (Y_{k},Y_{j_{\circ }})\leq \max\{\lambda (Y_{k},Y_{j_{\circ }}),\frac{1}{2}[\lambda (Y_{k},ϜY_{k})+\lambda (Y_{j_{\circ }},ϜY_{j_{\circ }})],\frac{1}{2}[\lambda (Y_{k},ϜY_{j_{\circ }})+\lambda (Y_{j_{\circ }},ϜY_{k})],M_{Ϝ}^{\varsigma }(Y_{k},Y_{j_{\circ }})\}\leq \max\{\lambda (Y_{k},Y_{j_{\circ }}),\frac{1}{2}[\lambda (Y_{k},Y_{k+1})+\lambda (Y_{j_{\circ }},Y_{j_{\circ }+1})],\frac{1}{2}[\lambda (Y_{k},Y_{j_{\circ }+1})+\lambda (Y_{j_{\circ }},Y_{k+1})],M_{Ϝ}^{\varsigma }(Y_{k},Y_{j_{\circ }})\}<\max\{\varepsilon +\frac{\delta^{\prime }}{2},\frac{1}{2}(\frac{\delta^{\prime }}{2}+\frac{\delta^{\prime }}{2}),\frac{1}{2}(\frac{\delta^{\prime }}{2}+\frac{\delta^{\prime }}{2}),\varepsilon +\delta^{\prime }\}<\max\{\varepsilon +\frac{\delta^{\prime }}{2},\delta^{\prime },\delta^{\prime },\varepsilon +\delta^{\prime }\}=\varepsilon +\delta^{\prime },$$

which implies by (3.2) that

(3.11) $$\rho (Y_{k},Y_{j_{\circ }})\lambda (ϜY_{k}.ϜY_{j_{\circ }})\leq \varepsilon .$$

However, considering the mapping *Ϝ* is triangular *ρ*-orbital admissible, in conjuction with (3.4), gives

$$\rho (Y_{n},Y_{n+1})\geq 1\;\mathrm{and}\;\rho (Y_{n+1},ϜY_{n+1})\geq 1\;\mathrm{implies}\;\rho (Y_{n},Y_{n+2})\geq 1,$$

and recursively, we get that

(3.12) $$\rho (Y_{n},Y_{k})\leq 1,$$

for all n,k∈N. Therefore from (3.11) and (3.12), we have

(3.13) $$\lambda (Y_{k+1},Y_{j_{\circ }+1})=\lambda (ϜY_{k},ϜY_{j_{\circ }})\leq \varepsilon .$$

Now, by triangle inequality together with (3.7) and (3.13), we get

$$\lambda (Y_{k+1},Y_{j_{\circ }})\leq \lambda (Y_{k+1},Y_{j_{\circ }+1})+\lambda (Y_{j_{\circ }+1},Y_{j_{\circ }})<(\varepsilon +\frac{\delta^{\prime }}{2}),$$

which means that, indeed $ϜY_{k}=Y_{k+1}\in A$.

**Case (2):** Suppose that

(3.14) $$\lambda (Y_{k},Y_{j_{\circ }})\leq \varepsilon .$$

Thus,

(3.15) $$\lambda (ϜY_{k},Y_{j_{\circ }})\leq \lambda (ϜY_{k},ϜY_{j_{\circ }})+\lambda (ϜY_{j_{\circ }},Y_{j_{\circ }})\leq \rho (Y_{k},Y_{j_{\circ }})\lambda (ϜY_{k},ϜY_{j_{\circ }})+\lambda (Y_{j_{\circ }+1},Y_{j_{\circ }})<\max\{\lambda (Y_{K},Y_{j_{\circ }}),\frac{1}{2}[\lambda (Y_{k},ϜY_{k})+\lambda (Y_{j_{\circ }},ϜY_{j_{\circ }})],\frac{1}{2}[\lambda (Y_{k},ϜY_{j_{\circ }})+\lambda (Y_{j_{\circ }},ϜY_{k})],M_{Ϝ}^{\varsigma }(Y_{k},Y_{j_{\circ }})\}+\lambda (Y_{j_{\circ }+1},Y_{j_{\circ }}),$$

where

$$M_{Ϝ}^{\varsigma }(Y_{k},Y_{j_{\circ }})={\left[\beta_{1}{\left(\frac{\lambda (Y_{k},Y_{k+1}),\lambda (Y_{j_{\circ }},Y_{j_{\circ }+1})}{\lambda (Y_{k},Y_{j_{\circ }})}\right)}^{\varsigma }+\beta_{2}{\left(\lambda (Y_{k},Y_{j_{\circ }})\right)}^{\varsigma }\right]}^{\frac{1}{\varsigma }}.$$

We must consider two subcases.

**(2a)**

$$\lambda (Y_{k},Y_{j_{\circ }})\geq \lambda (Y_{j_{\circ }},Y_{j_{\circ }+1}).\;\mathrm{Then},$$

$$M_{Ϝ}^{\varsigma }(Y_{k},Y_{j_{\circ }})={\left[\beta_{1}{\left(\frac{\lambda (Y_{k},Y_{k+1}),\lambda (Y_{j_{\circ }},Y_{j_{\circ }+1})}{\lambda (Y_{k},Y_{j_{\circ }})}\right)}^{\varsigma }+\beta_{2}{\left(\lambda (Y_{k},Y_{j_{\circ }})\right)}^{\varsigma }\right]}^{\frac{1}{\varsigma }}\leq {\left[\beta_{1}{\left(\lambda (Y_{k},Y_{k+1}\right)}^{\varsigma }+\beta_{2}{\left(\lambda (Y_{k},Y_{j_{\circ }})\right)}^{\varsigma }\right]}^{\frac{1}{\varsigma }}<{\left[\beta_{1}{\left(\frac{\delta^{\prime }}{2}\right)}^{\varsigma }+\beta_{2}{\left(\frac{\delta^{\prime }}{2}\right)}^{\varsigma }\right]}^{\frac{1}{\varsigma }}<{\left(\beta_{1}+\beta_{2}\right)}^{\frac{1}{\varsigma }}(\frac{\delta^{\prime }}{2}).$$

However, as $\delta^{\prime }=\min\{\delta^{\prime },\varepsilon ,1\}$, we obtain

$$M_{Ϝ}^{\varsigma }(Y_{k},Y_{j_{\circ }})<\frac{\varepsilon }{2},$$

and then

$$\lambda (Y_{k},Y_{j_{\circ }})<\max\{\lambda (Y_{k},Y_{j_{\circ }}),\frac{1}{2}[\lambda (Y_{k},Y_{k+1})+\lambda (Y_{j_{\circ }},Y_{j_{\circ }+1})],\frac{1}{2}[\lambda (Y_{k},Y_{j_{\circ }+1})+\lambda (Y_{j_{\circ }},Y_{k+1})],M_{Ϝ}^{S}(Y_{k},Y_{j_{\circ }})\}+\lambda (Y_{j_{\circ }+1},Y_{j_{\circ }})<\max\{\varepsilon ,\delta^{\prime },\delta^{\prime },\frac{\varepsilon }{2}\}+\frac{\delta^{\prime }}{2}=\varepsilon +\frac{\delta^{\prime }}{2},$$

which shows that $ϜY_{k}\in A$.

**(2b)**$\lambda (Y_{k},Y_{j_{\circ }})<\lambda (Y_{j_{\circ }},Y_{j_{\circ }+1})$. Then,

$$\lambda (ϜY_{k},Y_{j_{\circ }})\leq \lambda (Y_{k+1},Y_{k})+\lambda (Y_{k},Y_{j_{\circ }})<\frac{\delta^{\prime }}{2}+\frac{\delta^{\prime }}{2}<\varepsilon +\frac{\delta^{\prime }}{2}.$$

As a result, choosing some m,n∈N such that $m>n>j_{\circ }$, we can write

$$\lambda (Y_{m},Y_{n})\leq \lambda (Y_{m},Y_{j_{\circ }})+\lambda (Y_{j_{\circ }},Y_{n})<2\left(\varepsilon +\frac{\delta^{\prime }}{2}\right)<4\varepsilon ,$$

which lead us to

$$\lim_{m,n\to +\infty }\lambda (Y_{m},Y_{n})=0.$$

Consequently, in a complete metric space $\left\{Y_{m}\right\}$ is a Cauchy sequence. Finding a point u∈S such that $\lim_{m\to +\infty }Y_{n}=u$ is therefore possible. Additionally, because the mapping *Ϝ* is continuous we have

$$u=\lim_{m\to +\infty }{Ϝ}^{m+1}Y_{\circ }=\lim_{m\to +\infty }Ϝ({Ϝ}^{m}x_{\circ })=Ϝ(\lim_{m\to +\infty }{Ϝ}^{m}x_{\circ })=Ϝu,$$

which means that, u is a fixed point of *Ϝ*.

**Case (B):** Letting $Y=Y_{n-1}$ and $Z=Y_{n}=ϜY_{n-1}$ in (3.2), for the case ς=0, we obtain

(3.16) $$\lambda (Y_{n},Y_{n+1})\leq \rho (Y_{n-1},Y_{n})\lambda (ϜY_{n-1},ϜY_{n})<\max\{\lambda (Y_{n-1},Y_{n}),\frac{1}{2}[\lambda (Y_{n-1},ϜY_{n-1})+\lambda (Y_{n},ϜY_{n})],\frac{1}{2}[\lambda (Y_{n-1},ϜY_{n})+\lambda (Y_{n},ϜY_{n-1})],M_{Ϝ}^{\varsigma }(Y_{n-1},Y_{n})\},$$

where

$$M_{Ϝ}^{\varsigma }(Y_{n-1},Y_{n})={\left[\lambda (Y_{n-1},ϜY_{n-1})\right]}^{\beta_{1}}{\left[\lambda (Y_{n},ϜY_{n})\right]}^{\beta_{2}}={\left[\lambda (Y_{n-1},Y_{n})\right]}^{\beta_{1}}{\left[\lambda (Y_{n},Y_{n+1})\right]}^{\beta_{2}}.$$

Thus by taking (3.3) and (3.4) into account, we have

$$\lambda (Y_{n},Y_{n+1})\leq \rho (Y_{n-1},Y_{n})\lambda (ϜY_{n-1},ϜY_{n})<\max\{\lambda (Y_{n-1},Y_{n}),\frac{1}{2}[\lambda (Y_{n-1},ϜY_{n-1})+\lambda (Y_{n},ϜY_{n})],\frac{1}{2}[\lambda (Y_{n-1},ϜY_{n})+\lambda (Y_{n},ϜY_{n-1})],M_{Ϝ}^{\varsigma }(Y_{n-1},Y_{n})\}\leq \max\{\lambda (Y_{n-1},Y_{n}),\frac{1}{2}[\lambda (Y_{n-1},Y_{n})+\lambda (Y_{n},Y_{n+1})],\frac{1}{2}[\lambda (Y_{n-1},Y_{n+1})+\lambda (Y_{n},Y_{n})],M_{Ϝ}^{\varsigma }(Y_{n-1},Y_{n})\}.$$

Now, if we can find $n_{\circ }\in N$ such that $\lambda (Y_{n_{\circ }},Y_{n_{\circ }+1})\geq \lambda (Y_{n_{\circ }-1},Y_{n_{\circ }})$, we get

$$\lambda (Y_{n_{\circ }},Y_{n_{\circ }+1})<\max\{\lambda (Y_{n_{\circ }},Y_{n_{\circ }+1}),\frac{1}{2}[\lambda (Y_{n_{\circ }},Y_{n_{\circ }+1})+\lambda (Y_{n_{\circ }},Y_{n_{\circ }+1})],\;\;\;\;\frac{1}{2}[\lambda (Y_{n_{\circ }-1},Y_{n_{\circ }+1})],M_{Ϝ}^{\varsigma }(Y_{n_{\circ }-1},Y_{n_{\circ }})\}<\max\{\lambda (Y_{n_{\circ }},Y_{n_{\circ }+1}),\lambda (Y_{n_{\circ }},Y_{n_{\circ }+1}),\frac{1}{2}[\lambda (Y_{n_{\circ }-1},Y_{n_{\circ }+1})],\;\lambda (Y_{n_{\circ }},Y_{n_{\circ }+1})\}<\lambda (Y_{n_{\circ }},Y_{n_{\circ }+1}),$$

which is a contradiction. Therefore, $\lambda (Y_{n},Y_{n+1})<\lambda (Y_{n-1},Y_{n})$ for all n∈N, which means that $\left\{\lambda (Y_{n},Y_{n+1})\right\}$ is a decreasing sequence and eventually converges to some l≥0. Furthermore, since

$$M_{Ϝ}^{\varsigma }(Y_{n-1},Y_{n})={\left[\lambda (Y_{n-1},Y_{n})\right]}^{\beta_{1}}{\left[\lambda (Y_{n},Y_{n+1})\right]}^{\beta_{2}},$$

we get that

$$\lim_{n\to +\infty }\max\{\lambda (Y_{n-1},Y_{n}),\frac{1}{2}[\lambda (Y_{n-1},Y_{n})+\lambda (Y_{n},Y_{n+1})],\frac{1}{2}[\lambda (Y_{n},Y_{n+1})],M_{Ϝ}^{\varsigma }(Y_{n-1},Y_{n})\}=l.$$

Assuming that l>0, then $0<l<\lambda (Y_{n-1},Y_{n})$ and we can find δ>0 such that

$$l<\max\{\lambda (Y_{n-1},Y_{n}),\frac{1}{2}[\lambda (Y_{n-1},Y_{n})+\lambda (Y_{n},Y_{n+1})],\frac{1}{2}[\lambda (Y_{n},Y_{n+1})],M_{Ϝ}^{\varsigma }(Y_{n-1},Y_{n})\}=l+\delta .$$

By assuming ε=l in this manner, returns

$$l=\varepsilon <\max\{\lambda (Y_{n-1},Y_{n}),\frac{1}{2}[\lambda (Y_{n-1},Y_{n})+\lambda (Y_{n},Y_{n+1})],\frac{1}{2}[\lambda (Y_{n},Y_{n+1})],M_{Ϝ}^{\varsigma }(Y_{n-1},Y_{n})\}=\varepsilon +\delta ,$$

which implies that

$$\lambda (Y_{n-1},Y_{n})\leq \rho (Y_{n-1},Y_{n})\lambda (ϜY_{n-1},ϜY_{n})\leq \varepsilon =l,$$

which is a contradiction. We thus proved that

(3.17) $$\lim_{n\to +\infty }\lambda (Y_{n-1},Y_{n})=0.$$

We now assert that $Y_{n}$ is a Cauchy sequence. Firstly, we note that $\lim_{n\to +\infty }\lambda (Y_{n-1},Y_{n})=0$, we can find $j_{\circ }\in N$, such that

(3.18) $$\lambda (Y_{n-1},Y_{n})<\frac{\delta^{\prime }}{2},$$

for any $n\geq j_{\circ }$ where $\delta^{\prime }=\{\delta ,\varepsilon ,1\}$.

Using inductive reasoning, we shall demonstrate that the following relation

(3.19) $$\lambda (Y_{j\circ },Y_{j_{\circ }+m})<\varepsilon +\frac{\delta^{\prime }}{2}$$

holds, for any m∈N. In case of m=1,

$$\lambda (Y_{j_{\circ }},Y_{j_{\circ }+1})<\frac{\delta^{\prime }}{2}<\varepsilon +\frac{\delta^{\prime }}{2},$$

thus, (3.19) is accurate. Assuming that (3.19) holds for some *k*, we will now demonstrate that it also holds for k+1. In light of this, we have

$$M_{Ϝ}^{\varsigma }(Y_{j_{\circ }},Y_{j_{\circ }+1})={\left[\lambda (Y_{j_{\circ }},ϜY_{j_{\circ }})\right]}^{\beta_{1}}{\left[\lambda (Y_{j_{\circ }+k},ϜY_{j_{\circ }+1})\right]}^{\beta_{2}}={\left[\lambda (Y_{j_{\circ }},Y_{j_{\circ }+1})\right]}^{\beta_{1}}{\left[\lambda (Y_{j_{\circ }+k},Y_{j_{\circ }+k+1})\right]}^{\beta_{2}}<{\left(\frac{\delta^{\prime }}{2}\right)}^{\beta_{1}\beta_{2}}\leq \varepsilon +\frac{\delta^{\prime }}{2}.$$

Similar to Case(A), if $\lambda (Y_{j_{\circ }},Y_{j_{\circ }+1})<\varepsilon$, then by using (3.3) and applying the previously mentioned inequalities, we obtain

$$\varepsilon <\lambda (Y_{j_{\circ }},Y_{j_{\circ }+k})\leq \max\{\lambda (Y_{j_{\circ }},Y_{j_{\circ }+k}),\frac{1}{2}[\lambda (Y_{j_{\circ }},Y_{j_{\circ }+1})+\lambda (Y_{j_{\circ }+k},Y_{j_{\circ }+k+1})],\;\;\frac{1}{2}[\lambda (Y_{j_{\circ }},Y_{j_{\circ }+k+1})+\lambda (Y_{j_{\circ }},Y_{j_{\circ }+1})],M_{Ϝ}^{S}(Y_{j_{\circ }},Y_{j_{\circ }+k})\}<\max\{\frac{\delta^{\prime }}{2},\delta^{\prime },\delta^{\prime },\varepsilon +\frac{\delta^{\prime }}{2}\}=\varepsilon +\frac{\delta^{\prime }}{2},$$

which implies

$$\rho (Y_{j_{\circ }},Y_{j_{\circ }+k})\lambda (ϜY_{j_{\circ }},ϜY_{j_{\circ }+k})\leq \varepsilon .$$

Now, using (3.12), it follows that

$$\lambda (Y_{j_{\circ }},Y_{j_{\circ }+k+1})=\lambda (ϜY_{j_{\circ }+1},ϜY_{j_{\circ }+k})\leq \varepsilon ,$$

and then, by (3.19) we get

$$\lambda (Y_{j_{\circ }},Y_{j_{\circ }+k+1})\leq \lambda (Y_{j_{\circ }},Y_{j_{\circ }+1}+\lambda (Y_{j_{\circ }+1},Y_{j_{\circ }+k+1})<\frac{\delta^{\prime }}{2}+\varepsilon .$$

Therefore, (3.19) holds for (k+1). In the converse circumstance, if $\lambda (Y_{j_{\circ }},Y_{j_{\circ }+k})\leq \varepsilon$, again by triangle inequality, we obtain

$$\lambda (Y_{j_{\circ }},Y_{j_{\circ }+k+1})\leq \lambda (Y_{j_{\circ }},Y_{j_{\circ }+1})+\lambda (Y_{j_{\circ }+1},Y_{j_{\circ }+k+1})\leq \lambda (Y_{j_{\circ }},Y_{j_{\circ }+1})+\rho (Y_{j_{\circ }},Y_{j_{\circ }+k})\lambda (ϜY_{j_{\circ }},ϜY_{j_{\circ }+1})<\lambda (Y_{j_{\circ }},Y_{j_{\circ }+1})+\max\{\lambda (Y_{j_{\circ }},Y_{j_{\circ }+k}),\frac{1}{2}[\lambda (Y_{j_{\circ }},Y_{j_{\circ }+1})+\lambda (Y_{j_{\circ }+k},Y_{j_{\circ }+k+1})],\frac{1}{2}[\lambda (Y_{j_{\circ }},Y_{j_{\circ }+k+1})+\lambda (Y_{j_{\circ }},Y_{j_{\circ }+1})],\;\;\;M_{Ϝ}^{S}(Y_{j_{\circ }},Y_{j_{\circ }+k})\}<\frac{\delta^{\prime }}{2}+\max\{\frac{\delta^{\prime }}{2},\delta^{\prime },\delta^{\prime },(\varepsilon +\frac{\delta^{\prime }}{2})\}=\frac{\delta^{\prime }}{2}+\varepsilon +\frac{\delta^{\prime }}{2}=\varepsilon +\delta^{\prime }.$$

The induction argument is now complete. Consequently, $\left\{Y_{n}\right\}$ is a Cauchy sequence in *S*. Therefore, we can find an element u∈S satisfying Ϝu=u. □

In the above theorem, the continuity hypothesis of the mapping *Ϝ* can be substituted with the continuity of ${Ϝ}^{2}$ in the following manner.

Theorem 3.4 *Suppose that*Ϝ:S⟶S*forms a ρ-hybrid-Jaggi-Meir-Keeler- Zamfirescu type contraction such that*${Ϝ}^{2}$*is continuous. Then, Ϝ has a fixed point, provided that we can find*$Y_{\circ }\in S$*such that*$\rho (Y_{\circ },ϜY_{\circ })\geq 1$*.*

Proof Let $Y_{\circ }\in S$ such that $\rho (Y_{\circ },ϜY_{\circ })\geq 1$. For any n∈N, let $Y_{n}=ϜY_{n-1}$ define the sequence $\left\{Y_{n}\right\}$. Then, we can infer that $\left\{Y_{n}\right\}$ is a convergent sequence from Theorem 3.3. Letting $u=\lim_{m\to +\infty }Y_{n}$, we claim that u=Ϝu.

Given that the mapping ${Ϝ}^{2}$ is continuous,

$${Ϝ}^{2}u=\lim_{m\to +\infty }{Ϝ}^{2}Y_{n}=u.$$

To see this, we shall assume contrary to our claim, that is u≠Ϝu, then we have

$$M_{Ϝ}^{\varsigma }(u,Ϝu)=\left\{ \begin{array}{c} {\left[\beta_{1}{\left(\frac{\lambda (u,Ϝu)\lambda (Ϝu,{Ϝ}^{2}u)}{\lambda (u,Ϝu)}\right)}^{\varsigma }+\beta_{2}{\left(\lambda (u,Ϝu)\right)}^{\varsigma }\right]}^{\frac{1}{\varsigma }},\\ \;\;\;\;\;\;\;\;\;for\;\varsigma >0;\\ {\left(\lambda (u,Ϝu)\right)}^{\beta_{1}}{\left(\lambda (Ϝu,{Ϝ}^{2}u)\right)}^{\beta_{2}},\;for\;\varsigma =0, \end{array}\right.=\left\{ \begin{array}{c} {\left[\beta_{1}{\left(\frac{\lambda (u,Ϝu)\lambda (Ϝu,u)}{\lambda (u,Ϝu)}\right)}^{\varsigma }+\beta_{2}{\left(\lambda (u,Ϝu)\right)}^{\varsigma }\right]}^{\frac{1}{\varsigma }},\\ \;\;\;\;\;\;\;\;\;for\;\varsigma >0;\\ {\left(\lambda (u,Ϝu)\right)}^{\beta_{1}}{\left(\lambda (Ϝu,u)\right)}^{\beta_{2}},\;for\;\varsigma =0, \end{array}\right.=\left\{ \begin{array}{c} {\left[\beta_{1}{\left(\lambda (Ϝu,u)\right)}^{\varsigma }+\beta_{2}{\left(\lambda (u,Ϝu)\right)}^{\varsigma }\right]}^{\frac{1}{\varsigma }},\\ \;\;\;\;\;\;\;\;\;for\;\varsigma >0;\\ 0,\;for\;\varsigma =0, \end{array}\right.=\left\{ \begin{array}{c} {\left[\beta_{1}+\beta_{2}\right]}^{\frac{1}{\varsigma }}(\lambda (Ϝu,u)),\\ \;\;\;\;\;for\;\varsigma >0;\\ 0,\;for\;\varsigma =0. \end{array}\right.$$

If to the hypothesis of Theorem 3.3, we add the following assumption

ρ(u,v)≥1foranyu,v∈Fix(S),

then the mapping *Ϝ* has a unique fixed point.

To see this, we let u∈S be a fixed point of *Ϝ*. Suppose that we can find v∈S with Ϝu=u≠v=Ϝv, then,

**(i)** For ς>0,

$$M_{Ϝ}^{S}(u,Ϝu)={\left[\beta_{1}{\left(\frac{\lambda (u,Ϝu)\lambda (Ϝu,u)}{\lambda (v,Ϝv)}\right)}^{\varsigma }+\beta_{2}{\left(\lambda (u,v)\right)}^{\varsigma }\right]}^{\frac{1}{\varsigma }}={\left[\beta_{2}\right]}^{\frac{1}{\varsigma }}\lambda (u,v)\leq \lambda (u,v).$$

Therefore, if we take Y=u and Z=v in (3.3), we get a contradiction, since

$$\lambda (u,v)\leq \rho (u,v)\lambda (Ϝu,Ϝv)<\max\{\lambda (u,v),\frac{1}{2}[\lambda (u,Ϝu)+\lambda (v,Ϝv)],\frac{1}{2}[\lambda (u,Ϝv)+\lambda (v,Ϝu)],M_{Ϝ}^{\varsigma }(u,v)\}=\lambda (u,v).$$

**(ii)** For ς=0,

$$M_{Ϝ}^{\varsigma }(u,v)={\left[\lambda (u,Ϝu)\right]}^{\beta_{1}}{\left[\lambda (v,Ϝv)\right]}^{\beta_{1}}=0.$$

Therefore,

$$\lambda (u,v)\leq \rho (u,v)\lambda (Ϝu,Ϝv)<\max\{\lambda (u,v),\frac{1}{2}[\lambda (u,Ϝu)+\lambda (v,Ϝv)],\;\frac{1}{2}[\lambda (u,Ϝv)+\lambda (v,Ϝu)],M_{Ϝ}^{\varsigma }(u,v)\}<\max\{\lambda (u,v),0,\lambda (u,v),0\}=\lambda (u,v),$$

a contradiction. Consequently, if we can find a fixed point of the mapping *Ϝ*, under the assumption of the theorem, then it must be unique. □

Example 3.5 Let S=[0,+∞), λ:S×S⟶[0,+∞) be defined by λ(Y,Z)=|Y−Z|, then (S,λ) is a complete metric space. Define the mapping Ϝ:S⟶S by

$$ϜY=\left\{ \begin{array}{cc} \frac{1}{3}, & \text{if}\;Y\in [0,1]\\ \frac{1}{7}, & \text{if}\;Y>1. \end{array}\right.$$

Clearly, we can see that *Ϝ* is discontinuous at the point Y=1, but ${Ϝ}^{2}$ is a continuous mapping.

For Y∈[0,1],

$${Ϝ}^{2}Y=Ϝ(ϜY)=Ϝ\left(\frac{1}{3}\right)=\frac{1}{3},$$

for Y>1,

$${Ϝ}^{2}Y=Ϝ(ϜY)=Ϝ\left(\frac{1}{7}\right)=\frac{1}{3}.$$

Therefore,

$${Ϝ}^{2}Y=\left\{ \begin{array}{cc} \frac{1}{3}, & \text{if }Y\in [0,1]\\ \frac{1}{3}, & \text{if }Y>1. \end{array}\right.$$

Let the function ρ:S×S⟶[0,+∞) be defined by

$$\rho (Y,Z)=\left\{ \begin{array}{cc} Y^{2}+Z^{2}+1, & \text{if}\;Y,Z\in [0,1]\\ \ln(Y+Z), & \text{if}\;Y,Z\in (1,+\infty )\\ 1, & \text{if}\;Y=\frac{6}{7},Z=\frac{8}{7}\\ 0, & \text{otherwise}. \end{array}\right.$$

Choose $\beta_{1}=\frac{1}{5}$, $\beta_{2}=\frac{1}{3}$ and s=2. The mapping *Ϝ* is *ρ*-orbital admissible and satisfies Lemma 3.2 for any Y,Z∈[0.1], respectively for Y,Z∈(1,+∞). Taking into consideration the definition of the function *ρ*, we need to investigate further the case $Y=\frac{6}{7}$ and $Z=\frac{8}{7}$. So, we have

$$M_{Ϝ}^{\varsigma }(\frac{6}{7},\frac{8}{7})={\left[\frac{1}{5}{\left(\frac{\lambda (\frac{6}{7},Ϝ\frac{6}{7})\lambda (\frac{8}{7},Ϝ\frac{8}{7})}{\lambda (\frac{6}{7},\frac{8}{7})}\right)}^{2}+\frac{1}{3}{\left(\lambda (\frac{6}{7},\frac{8}{7})\right)}^{2}\right]}^{\frac{1}{2}}={\left[\frac{1}{5}{\left(\frac{\lambda (\frac{6}{7},\frac{1}{3})\lambda (\frac{8}{7},\frac{1}{7})}{\lambda (\frac{6}{7},\frac{8}{7})}\right)}^{2}+\frac{1}{3}{\left(\lambda (\frac{6}{7},\frac{8}{7})\right)}^{2}\right]}^{\frac{1}{2}}=\sqrt{\frac{121}{180}+\frac{4}{147}}=\sqrt{\frac{6169}{8820}}.$$

Therefore,

$$\rho \left(\frac{6}{7},\frac{8}{7}\right)\lambda \left(Ϝ\frac{6}{7},Ϝ\frac{8}{7}\right)=\frac{4}{21}<\sqrt{\frac{6169}{8820}}=\max\{\lambda \left(\frac{6}{7},\frac{8}{7}\right),\frac{1}{2}[\lambda \left(\frac{6}{7},\frac{1}{3}\right)+\lambda \left(\frac{80}{7},\frac{1}{7}\right)],\;\frac{1}{2}[\lambda \left(\frac{6}{7},\frac{1}{7}\right)+\lambda \left(\frac{8}{7},\frac{1}{3}\right)],M_{Ϝ}^{\varsigma }\left(\frac{6}{7},\frac{8}{7}\right)\}=\max\{\frac{1}{7},\frac{16}{21},\frac{16}{21},\sqrt{\frac{6169}{8820}}\}.$$

Moreover, since the mapping *Ϝ* satisfies (3.1), then taking

$$\delta (\varepsilon )=\left\{ \begin{array}{cc} 1-\varepsilon , & \text{for }\varepsilon <1\\ 1, & \text{for }\varepsilon \geq 1, \end{array}\right.$$

as a result, the conditions of Theorem 3.3 are met and $u=\frac{1}{3}$ is a fixed point of the mapping *Ϝ*.

It is interesting to note that the main result of [10] is not applicable to Example 3.5. To see, take Y=0 and Z=2. Let $\epsilon =\epsilon_{⁎}=\frac{4}{5}$ be given. Then, there exists a $\delta_{⁎}=2$ such that $\frac{4}{5}<2=\lambda (0,2)<\frac{4}{25}+2$, but $\lambda (Ϝ0,Ϝ2)=\frac{4}{21}>\epsilon_{⁎}$.

Definition 3.6 A mapping Ϝ:S⟶S is called a *ρ*-hybrid-Jaggi-Meir-keeler-Zamfirescu type contraction on *S* if for all distinct Y,Z∈S, we have

**(i)** For any given ε>0, there exists δ>0 such that

$$\varepsilon <\max\{\lambda (Y,Z),\frac{1}{2}[\lambda (Y,ϜY)+\lambda (Z,ϜZ)],\frac{1}{2}[\lambda (Y,ϜZ)+\lambda (Z,ϜY)],M_{Ϝ}^{\varsigma }(Y,Z)\}<\varepsilon +\delta ,$$

(3.20) ⇒ρ(Y,Z)λ(ϜY,ϜZ)≤ε;

**(ii)** whenever $M_{Ϝ}^{\varsigma }(Y,Z)>0$,

(3.21) $$\lambda (ϜY,ϜZ)<\max\{\lambda (Y,Z),\frac{1}{2}[\lambda (Y,ϜY)+\lambda (Z,ϜZ)],\frac{1}{2}[\lambda (Y,ϜZ)+\lambda (Z,ϜY)],M_{Ϝ}^{\varsigma }(Y,Z)\}.$$

Corollary 3.7 *Let*(S,λ)*be a complete metric space. Suppose the following conditions are satisfied:*

*(i)* *For every*ε>0,∃δ>0*and a mapping*$\rho :S\times S⟶R^{+}$*such that*
  (3.22) $$\varepsilon <\max\{\lambda (Y,Z),\frac{1}{2}[\lambda (Y,ϜY)+\lambda (Z,ϜZ)],\frac{1}{2}[\lambda (Y,ϜZ)+\lambda (Z,ϜY)]\}<\varepsilon +\delta ;$$
*(ii)* *Ϝ is a ρ-orbital admissible mapping;*
*(iii)* *we can find*$Y_{\circ }\in S$*such that*$\rho (Y_{\circ },ϜY_{\circ })\geq 1$*and*$\rho (Y_{\circ },{Ϝ}^{2}Y_{\circ })$*.*

*Then Ϝ has a fixed point in S.*

Proof Suppose that (3.22) holds, then

$$\varepsilon <\max\{\lambda (Y,Z),\frac{1}{2}[\lambda (Y,ϜY)+\lambda (Z,ϜZ)],\frac{1}{2}[\lambda (Y,ϜZ)+\lambda (Z,ϜY)]\}\leq \max\{\lambda (Y,Z),\frac{1}{2}[\lambda (Y,ϜY)+\lambda (Z,ϜZ),\frac{1}{2}[\lambda (Y,ϜZ)+\lambda (Z,ϜY)],\;\;\;\;\;M_{Ϝ}^{S}(Y,Z)]\}<\varepsilon +\delta ,$$

that is, *Ϝ* is a combinational contraction. Hence, all the assumptions of Theorem 3.3 are satisfied. Thus, *Ϝ* has a fixed point in *S*. □

## 4 An application to a Fredholm integral equation

The role of fixed point theory in the existence theory of differential and integral equations can be considered as having significant value given that almost all real-life problems can be reformulated as some forms of functional equations. Following the earliest application of the contraction mapping principle, many authors have applied fixed point techniques to analyze different models involving integral or differential equations. A few of such results can be found in [5], [11], [12], [16], [17] and some references therein.

In this section, one of our obtained results is applied to a specific class of nonlinear Fredholm integral equations. To begin, we establish a proposition that plays a key role in the subsequent discussion.

Proposition 4.1 *Suppose that*(S,λ)*is a metric space. Let*Ϝ:(S,λ)⟶(S,λ)*satisfy*

$$\rho (Y,Z)\lambda (ϜY,ϜZ)<k\max\{\lambda (Y,Z),\frac{1}{2}[\lambda (Y,ϜY)+\lambda (Z,ϜZ)],\frac{1}{2}[\lambda (Y,ϜZ)+\lambda (Z,ϜY)],M_{T}^{S}(Y,Z)\}$$

*for all*Y,Z∈S*and for some*k∈(0,1)*. Then Ϝ is a combinational contraction mapping.*

Proof Consider $\delta =(\frac{1}{k}-1)$, then we derive

$$\varepsilon \leq \max\{\lambda (Y,Z),\frac{1}{2}[\lambda (Y,ϜY)+\lambda (Z,ϜZ)],\frac{1}{2}[\lambda (Y,ϜZ)+\lambda (Z,ϜY)],M_{T}^{S}(Y,Z)\}<\varepsilon +\delta =\varepsilon +\left(\frac{1}{k}-1\right)=\frac{\varepsilon }{k}$$

and so for every Y,Z∈S, we obtain

$$k\varepsilon \leq k\max\{\lambda (Y,Z),\frac{1}{2}[\lambda (Y,ϜY)+\lambda (Z,ϜZ)],\frac{1}{2}[\lambda (Y,ϜZ)+\lambda (Z,ϜY)],M_{T}^{S}(Y,Z)\}<\varepsilon .$$

Using (3.22), we get λ(ϜY,ϜZ)<ε, with ρ(Y,Z)=1 for all Y,Z∈S. □

Throughout, C[a,b] denote the set of all continuous real-valued functions defined on [a,b]. Now, we try to obtain the criterion to ensure the existence of a solution for a type of nonlinear Fredholm integral equation.

Theorem 4.2 *Consider the nonlinear Fredholm integral equation*

(4.1) $$ϜY(t)=g(t)+\int_{a}^{b}P(t,s,Y(s))ds,$$

*for some*a,b∈R*,*g:[a,b]⟶R*and*$P:{\left[a,b\right]}^{2}\times R⟶R$*be two continuous maps. Also, assume that the subsequent properties hold:*

*(i)* Ϝ:C[a,b]⟶C[a,b]*is a continuous mapping;*
*(ii)* *we can find a constant*k∈(0,1)*satisfying*
  $$|P(t,s,Y(s))-P(t,s,Y(s))|\leq \frac{k\max\{|Y(t)-Z(t)|,\frac{1}{2}[|Y(t)-ϜY(t)|+}{b-a}\;\frac{\left|Z(t)-ϜZ(t)|],\frac{1}{2}[|Y(t)-ϜZ(t)|+|Z(t)-ϜY(t)|]\right\}}{b-a},$$
  *for all*t,s∈[a,b]*.*

*Then, Problem*(4.1)*has a unique solution in*C[a,b]*.*

Proof Supposed S=C[a,b]. Obviously, *S* is complete with respect to the metric $\lambda :S\times S⟶R^{+}$ defined as

$$\lambda (Y,Z)=\underset{t\in [a,b]}{\sup}|Y(t)-Z(t)|,$$

where Y,Z∈S. Now,

$$|ϜY(t)-ϜZ(t)|=|[g(t)+\int_{a}^{b}P(t,s,Y(s))ds]-[g(t)+\int_{a}^{b}P(t,s,Z(s))ds]|=|g(t)+\int_{a}^{b}P(t,s,Y(s))ds-g(t)-\int_{a}^{b}P(t,s,Z(s))ds|=|\int_{a}^{b}P(t,s,Y(s))ds-\int_{a}^{b}P(t,s,Z(s))ds|\leq \int_{a}^{b}|P(t,s,Y(s))ds-P(t,s,Z(s))ds|\leq \frac{k\max\{|Y(t)-Z(t)|,\frac{1}{2}[|Y(t)-ϜY(t)|+|Z(t)-ϜZ(t)|],}{b-a}\;\;\;\;\frac{\frac{1}{2}[|Y(t)-ϜZ(t)|+|Z(t)-ϜY(t)|]\}}{b-a}\int_{a}^{b}ds=k\max\{|Y(t)-Z(t)|,\frac{1}{2}[|Y(t)-ϜY(t)|+|Z(t)-ϜZ(t)|],\;\;\;\frac{1}{2}[|Y(t)-ϜZ(t)|+|Z(t)-ϜY(t)|]\}\leq k\max\{\lambda (Y,Z),\frac{1}{2}[\lambda (Y,ϜY)+\lambda (Z,ϜZ)],\frac{1}{2}[\lambda (Y,ϜZ)+\lambda (Z,ϜY)]\},$$

for all Y,Z∈S and t∈[o,∞]. Thus,

$$\underset{t\in [a,b]}{\sup}|ϜY(t)-ϜZ(t)|\leq k\max\{\lambda (Y,Z),\frac{1}{2}[\lambda (Y,ϜY)+\lambda (Z,ϜZ)],\frac{1}{2}[\lambda (Y,ϜZ)+\lambda (Z,ϜY)]\}$$

and therefore, for each Y,Z∈S,

$$\lambda (Y,Z)\leq k\max\{\lambda (Y,Z),\frac{1}{2}[\lambda (Y,ϜY)+\lambda (Z,ϜZ)],\frac{1}{2}[\lambda (Y,ϜZ)+\lambda (Z,ϜY)]\}.$$

This implies that *Ϝ* satisfies all the assumptions of Proposition 4.1, and hence *Ϝ* is a *ρ*-hydrid-Jaggi-Meir-Keeler-Zamfirescu type contraction. Therefore, by Theorem 3.3, Problem (4.1) has a solution in *S*. □

## 5 Conclusion

In this paper, we built on the foundational work of Banach and expanded on the ideas of Jaggi, Meir-Keeler, and Zamfirescu contractions to create a new approach to fixed-point theory. In particular, our research combined these concepts to develop a novel hybrid contraction, which has both theoretical and practical applications, particularly in solving integral equations.

## Consent for publication

Not applicable.

## Funding

This research work is funded by 10.13039/501100012639Prince Sultan University.

## CRediT authorship contribution statement

**Mohammed Shehu Shagari:** Writing – original draft, Formal analysis, Conceptualization. **Manzuma Mustapha:** Writing – review & editing, Methodology, Formal analysis. **Hala H. Taha:** Writing – review & editing, Methodology, Formal analysis. **Sarah Aljohani:** Writing – review & editing, Conceptualization. **Nabil Mlaiki:** Writing – review & editing, Writing – original draft, Methodology, Investigation, Conceptualization.

## Declaration of Competing Interest

The authors declare the following financial interests/personal relationships which may be considered as potential competing interests: S. Aljohani and N. Mlaiki report financial support was provided by 10.13039/501100012639Prince Sultan University. S. Aljohani and N. Mlaiki report financial support was provided by 10.13039/501100012639Prince Sultan University. If there are other authors, they declare that they have no known competing financial interests or personal relationships that could have appeared to influence the work reported in this paper.

## Data Availability

No data were used to support this research.
